# Parkinsonian Patients and Poor Awareness of Dyskinesias

**DOI:** 10.3389/fneur.2014.00032

**Published:** 2014-03-20

**Authors:** Sara Pietracupa, Anna Latorre, Alfredo Berardelli, Giovanni Fabbrini

**Affiliations:** ^1^Department of Neurology and Psychiatry, Sapienza University of Rome, Rome, Italy; ^2^IRCCS Neuromed, Pozzilli, Italy

**Keywords:** Parkinson disease, levodopa-induced dyskinesias, awareness of self, anosognosia, dorsolateral prefrontal cortex

The question of whether the awareness of levodopa-induced dyskinesias (LID) is reduced, or altogether missing, in patients with Parkinson’s disease (PD) has been attracting growing interest. Why is this topic important? Firstly, as studies addressing the efficacy of drugs on LID employ patients’ motor diaries as an outcome measure, poor LID self-awareness might interfere with data collection in clinical trials; secondly, poor LID self-awareness may result in increased doses of dopaminergic drugs, which could in turn be associated with an enhanced risk of side effects such as LID. Lastly, understanding this phenomenon may shed light on some pathophysiological aspects of LID in PD.

The few studies conducted so far concluded that at least a proportion of PD patients are either partially or totally unaware of the presence of LID. These studies used different methods to ascertain LID awareness as well as different patient populations. Consequently, several hypotheses have been postulated to explain poor self-awareness of LID, which suggest that several possible mechanisms may be implicated.

An impairment in the experience of moving abnormally was suggested by Vitale et al. (1) in a pilot study conducted on PD patients (and on patients with Huntington disease). The authors found that poor LID self-awareness was more marked in PD patients with mild LID, and therefore suggested that poor LID self-awareness merely reflects the fact that when LID are mild, their interference with normal activities is limited and patients tend to underestimate them. These results, however, were not confirmed in the study conducted by Sitek et al. (2), who instead observed, by means of a video protocol, that poor self-awareness of LID was more pronounced in patients with longer symptom duration (and therefore with possibly more severe LID). This finding reported by Sitek et al. (2) thus suggests that other mechanisms are likely to play a role in the phenomenon of poor self-awareness of LID. Amanzio et al. (3) believed that factors linked to the cognitive domain might be involved. In a well planned study, they investigated whether awareness of different movement disorders in cognitively intact PD patients differs in the *on* and *off* states. The results of their study revealed a significant difference between awareness of LID measured in the *on* state and awareness of hypo-bradykinesia assessed in the *off* state. In particular, while 22 of the 25 patients enrolled in their study displayed a reduced awareness of LID, a reduced awareness of hypo-bradykinetic movement disorders was found in only 6 of the 25 patients. On the basis on these findings, the authors suggested that dopaminergic therapy may, by stimulating mesocortical–limbic pathways, exert a detrimental effect on the function of the orbitofrontal and cingulated frontal–subcortical loops, thereby contributing to a poor self-awareness of LID. Supporting this view, recent papers indeed showed that frontal cortex studied by advanced neuroimaging techniques differ between patients with or without LID. In one study, PD patients with LID had significant overactivity in the supplementary motor area and underactivity in the right inferior prefrontal gyrus during execution of motor tasks when compared with PD patients without LID (4). In addition, in another study cortical thickness analysis revealed a pronounced increase of thickness in the right inferior frontal sulcus in PD patients with LID with respect to PD patients without LID (5). The hypothesis of Amanzio et al. is not, however, supported by the observations of other authors, who found that poor self-awareness is not only associated with LID, but also with motor symptoms assessed in the *off* state, and that awareness of *off* motor symptoms improved, at least in part, during the *on* state following dopaminergic stimulation (1, 6, 7). Maier et al. (7) also found that the severity of impairment of motor self-awareness was unrelated to dopamine-dependent executive functioning. These authors also reported that impaired awareness of abnormal movements (and therefore of LID) was significantly associated with the postural instability gait disorder phenotype of PD to a greater extent than with the hyperkinetic phenotype. Although the postural instability gait disorder phenotype is frequently associated with cognitive decline, it is noteworthy that the severity of the impaired awareness of movement in the study by Maier et al. (7) was not correlated with either disease duration or cognitive outcome. Other authors have proposed alternative or complementary hypotheses. For example, one noteworthy finding comes from the work by Jenkinson et al. (8), who highlighted the fact that normal motor awareness entails a correct comparison of intended vs. actual movement, and predicted in their work that anosognosia of LID in PD arises from a failure to detect discrepancies between intended movement and visual feedback. To test their hypothesis, they used a mirror to reverse the expected visual consequence of an executed movement. PD patients with poor self-awareness of LID did not detect any differences between congruent and incongruent movement, whereas non-anosognosic PD patients and healthy volunteers reported incongruent movement as being stranger than congruent movement. The findings of Jenkinson et al.’s (8) work thus support the hypothesis that normal motor awareness entails a comparison of intended and actual movement. Indeed, the intactness of the comparator in non-anosognosic PD patients and healthy volunteers is demonstrated by the finding that incongruent movement was reported to be stranger than congruent movement by both these groups, as well as by the fact that there were no significant differences between non-anosognosic PD patients and healthy volunteers. These results are in keeping with the hypothesis of a breakdown of the comparator mechanism in PD patients with anosognosia. According to this hypothesis, if reduced or lack of awareness of LID in PD is a form of anosognosia, then a dysfunction of the right hemisphere is presumably involved. If we bear in mind that PD is usually characterized by asymmetric motor involvement, this paradigm may prove useful as a means of verifying whether patients with more severe symptoms on the left side of the body suffer from a greater degree of anosognosia of LID than patients with more severe symptoms on the right side of the body. Indeed, dysfunction of the right hemisphere seems to contribute to impaired self-awareness of motor symptoms. By studying non-demented PD patients who were tested prior to a unilateral pallidotomy, Leritz et al. (6) found not only that PD patients as a group rated themselves as being less impaired than their caregivers did, but also that PD patients with right-sided symptoms (left pallidotomy patients) rated themselves as being more impaired on two ADL measures than patients with left-sided symptoms (right pallidotomy patients). In other words, patients with more severe symptoms on the left side of the body were less aware of their motor and functional deficit than patients with more severe symptoms on the right side of the body. In a recent work, we investigated awareness of LID in 30 PD patients who had no cognitive dysfunction (9). In that study, which was based on a video protocol, we initially found that 23.3% of the patients investigated were unaware of the presence of their LID. However, when patients were asked to recognize LID while watching video-recordings of themselves, most of them recognized their own LID. Moreover, the same patients recognized LID in video-recordings of reference PD patients. None of the clinical variables (e.g., age, duration of symptoms, severity of disease, duration, and dose of dopaminergic drugs) or neuropsychological variables we took into consideration correlated with poor self-awareness of LID. The only clinical feature that did correlate with poor self-awareness of LID was a prevalence of motor symptoms on the left side of the body. These results led us to hypothesize that LID unawareness is indeed predominantly a form of anosognosia. Might anosognosia of LID be another form of anosognosia due to right subcortical lesions, as has been reported in patients with hemichorea/hemiballism following contralateral infarction of the caudate nucleus (10)? It is more likely that PD patients with left hemi-body involvement are less aware of LID than patients with right hemi-body involvement because of an abnormal interaction in the complex network involving the posterior parietal cortex, the supplementary motor area, the premotor cortex, and the dorsolateral prefrontal cortex, all of which are regions that play a role in the awareness of voluntary movements (11, 12).

Awareness, in general, may also be linked to network activity, as demonstrated recently by Ham et al. (13) who investigated whether self-awareness deficits are associated with network dysfunction after traumatic brain injury. In this study, resting-state and event-related functional magnetic resonance imaging showed that neural activity within the fronto-parietal control network and the dorsal anterior cingulate cortex was abnormal in patients with impaired self-awareness. It could be worth exploring by means of resting-state and event-related functional magnetic resonance whether network dysfunction is also present in PD patients with poor awareness of LID.

Another question that warrants consideration is whether LID awareness varies depending on the body part examined. In this regard, we observed that PD patients are less aware of LID in the trunk (9). Although there is no clear explanation for this finding, some neurophysiological observations may help to shed light on the issue. In a noteworthy experimental study, Wright et al. (14) demonstrated that PD patients control the direction of axial twisting in both the hips and trunk less accurately than normal subjects. Another interesting observation that emerged from the study by Wright et al. (14) was that the proprioceptive deficit in the trunk may be exacerbated by levodopa administration. It is tempting to speculate, therefore, that mechanisms underlying this body site-specific poor awareness of LID may be due to the complex interplay between anosognosia and the impairment in axial kinesthesia observed in PD patients (14). Supporting this hypothesis is the observation that PD patients may also display poor awareness of other trunk abnormalities, such as bent trunk (camptocormia) and lateral deviation of the trunk (Pisa syndrome) (15). Poor awareness of LID in the trunk may be of considerable relevance in PD patients because it might contribute to the lack of control over balance and worsen the postural instability of PD patients, thereby predisposing patients in more complicated stages of the disease to falls during the *on* phases. Although much has yet to be understood regarding the intriguing phenomenon of poor motor and LID awareness in PD and other movement disorders, the studies we have analyzed in this brief review may be considered a starting point.
